# Gene-based analysis of genes related to neurotrophic pathway suggests association of *BDNF* and *VEGFA* with antidepressant treatment-response in depressed patients

**DOI:** 10.1038/s41598-018-25529-y

**Published:** 2018-05-03

**Authors:** Chung-Feng Kao, Yu-Li Liu, Younger W.-Y. Yu, Albert C. Yang, Eugene Lin, Po-Hsiu Kuo, Shih-Jen Tsai

**Affiliations:** 1 Department of Agronomy, National Chung Hsing University, Taichung, Taiwan; 2 Center for Neuropsychiatric Research, National Health Research Institutes, Miaoli County, Taiwan; 3 Yu’s Psychiatric Clinic, Kaohsiung, Taiwan; 4 Department of Psychiatry, Taipei Veterans General Hospital, Taipei, Taiwan; 5 Division of Psychiatry, National Yang-Ming University, Taipei, Taiwan; 6 Division of Interdisciplinary Medicine and Biotechnology, Beth Israel Deaconess Medical Center/Harvard Medical School, Boston, MA 02215 USA; 7 Institute of Brain Science, National Yang-Ming University, Taipei, Taiwan; 8 Graduate Institute of Biomedical Sciences, China Medical University, Taichung, Taiwan; 9 Department of Electrical Engineering, University of Washington, Seattle, WA 98195 USA; 10 Department of Public Health, Institute of Epidemiology and Preventive Medicine, National Taiwan University, Taipei, Taiwan

## Abstract

It is well established that brain-derived neurotrophic factor (BDNF) signaling pathway plays a key role in the pathophysiology of major depressive disorder (MDD) and in therapeutic mechanisms of antidepressants. We aim to identify genetic vairiants related to MDD susceptibility and antidepressant therapeutic response by using gene-based association analysis with genes related to the neurotrophic pathway. The present study investigated the role of genetic variants in the 10 neurotrophic-related genes (*BDNF*, *NGFR*, *NTRK2*, *MTOR*, *VEGFA*, *S100A10*, *SERPINE1*, *ARHGAP33*, *GSK3B*, *CREB1*) in MDD susceptibility through a case-control (455 MDD patients and 2,998 healthy controls) study and in antidepressant efficacy (n = 455). Measures of antidepressant therapeutic efficacy were evaluated using the 21-item Hamilton Rating Scale for Depression. Our single-marker and gene-based analyses with ten genes related to the neurotrophic pathway identified 6 polymorphisms that reached a significant level (*p*-value < 5.0 × 10^−3^) in both meta- and mega-analyses in antidepressant therapeutic response. One polymorphism was mapped to *BDNF* and 5 other polymorphisms were mapped to *VEGFA*. For case-control association study, we found that all of these reported polymorphisms and genes did not reach a suggestive level. The present study supported a role of *BDNF* and *VEGFA* variants in MDD therapeutic response.

## Introduction

Depression is a severe mental disorder and the leading cause of disabilities worldwide^1^. The reported prevalence throughout the world of depressive episodes is 1,607 per 100,000 per year for males and 2,552 per 100,000 per year for females^2^. Currently, the main medical treatment for depression is antidepressant medication. The selective serotonin reuptake inhibitors (SSRIs), including fluoxetine, sertraline, fluvoxamine, paroxetine and citalopram, are a popular family of antidepressants frequently prescribed at present. However, as with all antidepressant treatments, about 30–40% of major depressive disorder (MDD) patients do not respond sufficiently to SSRIs^3^. As evidence from earlier studies had indicated, genetic factors may play important roles in antidepressant responses^4,5^, and pharmacogenetic SSRI studies have attempted to identify genetic variants which predict antidepressant treatment response. Pharmacogenetics is the study of variability in drug response due to heredity, generally focusing on polymorphisms of genes related to the drug metabolizing-enzyme, drug action or disease pathophysiology^6^.

For candidate genes related to antidepressant therapeutic action, brain-derived neurotrophic factor (BDNF) has been a main focus of antidepressant pharmacogenetic research. BDNF, a member of the neurotrophin family, is a small dimeric protein widely expressed in adult mammalian brains with the highest levels found in the hippocampus^7^. BDNF plays a key role in the regulation of neuronal survival, differentiation, growth, and apoptosis by binding to two types of receptors; namely, tyrosine kinase B (trkB; encoded by the *NTRK2* gene) receptor and the p75 neurotrophin receptor (p75NTR; encoded by the *NGFR* gene). Earlier animal studies have demonstrated that stress of immobilization can lower BDNF mRNA levels in the hippocampus and other brain regions^8^. The role of BDNF in depression treatments was first revealed in research conducted by Nibuya and colleagues, where long-term administration of several types of antidepressants, including SSRIs, increases in BDNF expression in the rat hippocampus^9^. Furthermore, centrally administered BDNF produces antidepressant-like activities in animal models of depression^10^. In humans, post-mortem studies demonstrated an increase in BDNF immunoreactivity in the hippocampus of MDD subjects treated with antidepressant medication at the time of death, compared with untreated controls^11^. Many clinical studies also found that BDNF levels were significantly lower in MDD patients than in controls, and decreased serum levels of BDNF in MDD patients recovered to normal levels associated with the recovery of depression after treatment with antidepressant medication^12^. The above findings suggest that BDNF may be implicated in the pathogenesis of MDD and in antidepressant actions^13^, and may be a good candidate gene for antidepressant pharmacogenetic study.

The human *BDNF* gene has been mapped to chromosome 11p13. A common single nucleotide polymorphism (SNP) consisting of a missense change (G196A), producing a non-conservative amino acid change (valine to methionine), has been identified in the coding exon of the *BDNF* gene at position 66 (Val66Met, rs6265). The replacement of 66Val by 66Met disrupts cellular processing, trafficking, and activity-dependent secretion of BDNF^14^. In our 2003 study on 110 MDD patients, we examined the association between the *BDNF* Val66Met polymorphism and response to 4-week antidepressant (fluoxetine) treatment^15^. We found a trend showing better therapeutic response for the Val/Met-heterozygote patients in comparison to those bearing the homozygote (Val/Val or Met/Met). While similar findings have been reported in some of the subsequent studies, other studies found a better response in patients carrying the Met variant^16,17^. With the importance of BDNF in antidepressant therapeutic mechanism, *BDNF* gene has been the focus of antidepressant pharmacogenetic studies. Most of the *BDNF*-antidepressant pharmacogenetic studies investigated only the *BDNF* Val66Met polymorphism which may overlook other *BDNF* polymorphisms. Furthermore, a single gene may play only a small part in the antidepressant therapeutic response. Genes related to the BDNF function may interact with *BDNF* in response to antidepressant treatment.

Several genes related to neurotrophic pathway have also been implicated in the mechanisms underlying depression and drug action. For example, we have demonstrated that the *NTRK2* genetic variants interact with the *BDNF* Val66Met polymorphism contributing to the risk of geriatric depression^18^. One missense *NGFR* polymorphism (S250L) showed association with antidepressant therapeutic response^19^.We found that polymorphisms in the *GSK3B* gene (encoding glycogen synthase kinase-3 beta protein), an important component in the BDNF pathway, were associated with the antidepressant therapeutic response^20^. We also found that genetic variants in plasminogen activator inhibitor type 1 gene (encoded by the *SERPINE1* gene), which is involved in the cleavage of pro-BDNF to mature BDNF in the brain, are related to antidepressant therapeutic response and depression susceptibility^20^. Vascular endothelial growth factor (VEGF) is known to play a role in the process for neuroprotection and neurogenesis, and may be involved in the pathogenesis of some neurological disorders^21^. VEGF is encoded by *VEGFA*. S100 calcium-binding protein A10 (protein encoded by the *S100A10*), also known as p11, has a possible role in major depression’s pathophysiology^22^ and may also be involved in the cleavage of proBDNF to BDNF^23^. ARHGAP33 is a new type of regulator for the intracellular trafficking of TrkB signaling and is essential for synapse development. Dysfunction of this mechanism may be a new molecular pathology of neuropsychiatric disorders^24^. CREB protein (encoded by *CREB1*), a cellular transcription factor, plays a crucial role in turning on the BDNF gene^25^. Mammalian target of rapamycin (mTOR protein encoded by *MTOR*), a large serine/threonine kinase, regulates the initiation of protein translation in the body. A clinical study has found that ketamine produces rapid antidepressant effects in depressive patients^26^. An animal study demonstrated that ketamine stimulates AMPA receptor transmission and activates BDNF/TrkB-Akt/ERK-mTOR signaling cascades, leading to a sustained increase in synaptic protein synthesis and strengthening of synaptic plasticity^27^.

Pharmacogenomics, which is derived from genome-wide association (GWA) studies and pharmacogenetics, are proving to be increasingly useful in personalized medicinal research. Despite the progress from single SNP studies to GWA studies in antidepressant treatment response, results were not as expected as they were often inconsistent^28^. GWA studies have a much lower power when the number of SNPs increases and the SNPs are correlated, especially when their effect sizes are small^29^. Gene-based analysis is the SNP-based method, in which each SNP is tested for association, and multiple testing corrections based on the Bonferroni procedure are applied to control the type-I error rate. The concept of a gene-based association test has been broadly applied to pharmacogenomics studies^30^. The most important merit of SNP/gene-based analysis for pharmacogenomic studies over GWA studies is that we can target the genotyping of SNPs/genes efficiently provided the mechanisms of drug action are known. These selected SNPs/genes are connected with treatment responses, which can help in predicting the progress of a disease and use to the selection of targeted therapies in pharmacogenetic studies^31^. The strategy of SNP/gene-based analysis for pharmacogenetic studies provides clinicians to better use of knowledge of a list of genetic susceptible loci to link candidate genes and clinical drug related responses in order to make a rational treatment decision^32^. In this study, we conducted gene-based as well as single marker analyses in 10 genes related to the BDNF signaling pathway to identify genetic variation that may affect MDD risk or antidepressant therapeutic response.

## Materials and Methods

### Samples

We recruited 455 patients, 268 from NHRI (The National Health Research Institutes) and 187 from TVGH (Taipei Veterans General Hospital), who met DSM-IV criteria for major depressive disorder. The diagnosis was made by board-certified psychiatrists who interviewed patients and family members, and obtained records where possible. All subjects were Taiwanese. Subjects were part of the International SSRI Pharmacogenomics Consortium (ISPC) project, which included seven member sites from five countries^33^. Other inclusion criteria were minimum baseline score of 14 on the 21-item Hamilton Rating Scale for Depression (HRSD), and presence of depressive symptoms for at least two weeks before entry into the study without antidepressant treatment during that period (patients were fresh cases or had quit antidepressants for more than 2 weeks). Exclusion criteria were additional current DSM-IV Axis I diagnoses (including substance abuse, generalized anxiety disorders, panic disorders, or obsessive compulsive disorders), personality disorders, pregnancy, recent suicide attempt, and major medical and/or neurological disorders.

These patients were treated with SSRIs, which include escitalopram (38.5%), paroxetine (38.5%), fluoxetine (18.3%) and citalopram (4.8%). Treatment was initiated with a recommended starting dose (for example, the escitalopram dose was 10 mg/day in the beginning), and based on the clinical response after 2-week treatment the investigator could adjust the antidepressant dosage. The final mean antidepressant doses were 12.0 mg/day for escitalopram, 16.9 mg/day for paroxetine, 20.0 mg/day for fluoxetine and 19.0 mg/day for citalopram.

All participants were evaluated using the 21-item HRSD to measure depression severity. Patients were assessed repeatedly at baseline and week 2, 4, and 8. All raters for the HRSD received the same investigators’ training module. For the evaluation of the NHRI subjects, the inter-rater reliability coefficient was consistent, with a value of 0.9. The TVGH patients were evaluated by a senior psychiatrist (YWY).

Healthy controls were drawn from the Taiwan Biobank with a Taiwanese Han ancestry^34^, consisted of 2,998 participants in the current study (nearly equal proportions of males and females), whom were interviewed by trained interviewers with standard questionnaires to collect information of demographic variables, life-style and physical health conditions as well as clinical data. All controls were found to have no definite diagnosis of any major medical or mental illnesses, they underwent genotyping at the genome-wide level and were thus treated as controls in the current study.

Experiments were conducted in accordance with the Declaration of Helsinki and approved by the Institutional Review Board of Taipei Veterans General Hospital (VGHIRB No.: 2014-06-001B). Written informed consents were obtained from all participants ensuring adequate understanding of the study.

### Measurement

We used four repeated phenotypes to measure different treatment responses, including remitted, response, and stem-depressed. First, we calculated the sum score of 21-item HRSD at week 8, and recoded as 1 if the sum score greater than 7 and 2 (i.e., *remitted*) otherwise. Second, we calculated the percentage change of HRSD (i.e., %ΔHRSD), and recoded the results as 1 if the percentage change greater than −50% and 2 (i.e., *response*) otherwise. Third, we also used the percentage change of HRSD as a treatment-response index. Last, we used the score of ‘depressed mood’ item to measure depression, and recoded the results as 1 if the score greater than or equal to 3 and 2 (i.e., *stem-depressed*) otherwise. For detailed definitions of treatment-response phenotypes, please refer to Lin *et al*.^35^.

### Genotyping data and quality controls

All participants were genotyped using Illumina HumanOmniExpressExome BeadChips in the International SSRI Pharmacogenomics Consortium. A total of 455 subjects were genotyped with 951,123 SNPs. We selected 10 SSRIs treatment-responses or depression related candidate genes (*BDNF*, *NGFR*, *NTRK2*, *MTOR*, *VEGFA*, *S100A10*, *SERPINE1*, *ARHGAP33*, *GSK3B*, *CREB1*), which are related to the neurotrophic pathway. A total of 1,251 and 2,656 SNPs were mapped into the 10 selected candidate genes in mega- and meta-analysis, respectively.

Quality control procedures were done firstly with each individual, including sample quality, kinship and population stratification. We first check plate-wise genotyping biases. Samples with plate pass rate greater than 97% were retained in the analysis. A total of 18 samples (11 from NHRI and 7 from TVGH) were removed during this step. Second, we checked the inbreeding coefficient and identity by state (IBS), so that samples with strong kinship were eliminated. In total, 9 individuals (4 from NHRI and 5 from TVGH) were removed from the samples due to having similar measures which are far away from the clustering. Third, we used a multidimensional scaling analysis on the genome-wide IBS pairwise distance to eliminate samples with outliers. Our results showed that none was away from the clustering on the scatter plot. Finally, 7 patients treated with sertraline (SSRI) and venlafaxine (SNRI) were excluded from the analysis. As a result, 421 (mean age of 43.7 years and 71.3% of females) MDD patients were retained.

We also performed quality control procedures for markers. We removed markers which failed the Hardy-Weinberg tests with a *p*-value less than 0.0001, genotype missing rate greater than 5%, minor allele frequency (MAF) smaller than 0.05, or ones with bad calling in clustering. As a result, a total of 647,030 SNPs in the samples were retained for imputation. The genotyping call rate was 99.9% for all subjects.

### Imputation and gene mapping

Imputation was carried out using IMPUTE2 v3^36^, with haplotype reference panels released in March/April 2012 from the 1000 Genomes Project on the basis of HapMap build 37 (https://mathgen.stats.ox.ac.uk/impute/data_download_1000G_phase1_integrated_SHAPEIT2.html). Only imputed SNPs with high genotype information content (i.e. IMPUTE info score > 0.5) were used in association analyses. In total, 30,040,257 SNPs were imputed with high confidence for each individual in the samples. Following the same quality control procedures for markers, a total of 4,241,701 SNPs were retained for analyses. Gene-mapping was conducted using 50 kb upstream and downstream of the gene boundaries.

### Multiple imputation method for missing phenotypic data

To deal with the issue of missing data, we used multiple imputation approach to account uncertainty of the imputed data. We first created multiple-imputed datasets, by fitting regression models based on observed complete data from other variables to predict missing data multiple times (number of iterations was set to 30 by default) to account for uncertainty. Second, we performed standard statistical analyses on each of imputed datasets. Third, we pooled the results of each of these analyses to produce one set of results. The method of multiple imputation overcame the limitations of single imputation (e.g. LOCF) to provide more realistically estimated precision and less biased results. This approach is beneficial with several advantages, including unbiased parameter estimates, robust departures from normality assumptions, adequate results when sample size is small or high rates of missing data, computationally simple, and tractable solution to missing data problems.

### Genetic association analyses

We performed single-marker association and gene-based association tests for treatment-response phenotypes. Both linear and logistic regression analysis were conducted with additive genetic model. All models were adjusted for gender and age to correct for different distributions in gender and age across treatment-response phenotypes in MDD patients.

Gene-based association analyses were conducted to obtain gene-level empirically significant estimations. Information from a set of SNPs (association *p*-value < 0.1 by default) within a gene was aggregated. To account for the linkage disequilibrium (LD) among markers, only SNPs having *r*^2^ < 0.5 with each other were retained for each gene. We also fitted both linear and logistic regression models to perform gene-based association tests. Empirical *p*-values were calculated with 50,000 permutations.

To combine individual association results (i.e. *p*-values) from the two samples (i.e., NHRI and TVGH), meta-analysis was applied using the inverse Gamma model with a shape parameter (α) of 1, that is, the Fisher’s method^37^ to summarize association information across the two samples. Alternatively, we conducted association analyses to an integrated database of NHRI and TVGH. We adopted a method of LD adjusted multiple testing correction developed by Duggal *et al*.^38^ to account for the interdependence among SNPs to balance between false-negative and false-positive findings. Only SNPs and genes with *p*-values less than 5.0 × 10^−4^ (or 5.0 × 10^−3^) and 5.0 × 10^−2^ (or 1.0 × 10^−2^), either in mega-analysis or in meta-analysis, were considered to be significant (or suggestive), and were reported in single-marker association analysis and gene-based association analysis, respectively. Additionally, we conducted a case-control (455 MDD patients and 2,998 healthy controls) study using SNP-based and gene-based association analyses to investigate whether the treatment-response associated loci were also MDD susceptible loci.

All aforementioned analyses were conducted with R version 3.0.2, PLINK version 1. 90b3.37 64-bit, and haploview version 4.1. Additionally, to explore the potential roles of these six SNPs as expression quantitative trait locus (eQTL), we used HaploReg (http://compbio.mit.edu/HaploReg) to search gene regulation databases.

## Results

Among 428 subjects, 394 (92.06%) cases were observed at all four time points; 30 cases (7.02%) were missing at just one time-point of follow-up; and the remaining 4 cases (0.92%) were missing at two or more time-points of follow-up. Table 1 exhibits detailed patterns of missing data in phenotypes across different time points, indicating the issue of incomplete data. Here we conducted multiple imputation method to impute these missing data using available observed data, such that our data are complete. Furthermore, we excluded 7 subjects (4 treated with sertraline and 3 with venlafaxine) from the analysis. As a result, 421 MDD patients were retained for the following analyses. Table 2 represents summary statistics for treatment-response phenotypes. All three treatment-response phenotypes (remitted, response, and stem-depressed) showed significant difference (*p*-value < 2.2 × 10^−16^) between treatment responders and treatment non-responders. We found that 373 (88.6%) treatment-responders had moderate or less depressed mood compared to 48 (11.4%) treatment non-responders who had moderately severe or severe depressed mood. No gender difference was observed among all treatment-response phenotypes.

**Table 1:** The patterns of missing data in phenotypes (N = 428).

Time point	Missing data patterns								# of missing observation (%)
Baseline	Observed	Observed	Observed	Observed	Observed	Observed	Observed	Observed	0
Follow-up (week 2)	Observed	**Missing**	Observed	Observed	**Missing**	**Missing**	Observed	**Missing**	14 (3.27%)
Follow-up (week 4)	Observed	Observed	**Missing**	Observed	**Missing**	Observed	**Missing**	**Missing**	12 (2.80%)
Follow-up (week 8)	Observed	Observed	Observed	**Missing**	Observed	**Missing**	**Missing**	**Missing**	13 (3.04%)
# of cases	394	10	10	10	1	2	0	1	428
(%)	(92.06%)	(2.34%)	(2.34%)	(2.34%)	(0.23%)	(0.46%)	(0.00%)	(0.23%)	

^a^Missing represents missing value in all the 21-item Hamilton Rating Scale for Depression.

**Table 2:** Summary statistics for treatment-response phenotypes.

Treatment-response phenotypes	Mean (s.d.)/N (%)		Gender difference p-value	Treatment responder				Treatment non-responder				Difference test (p-value)
	Male (N = 121)	Female (N = 300)		N	(%)	Mean	s.d.	N	(%)	Mean	s.d.	
Remitted, Score	10.36 (6.02)	11.01 (6.24)	0.32	139	(33.02)	4.46	1.93	282	(66.98)	13.96	5.03	**<2.2 × 10** ^**−16**^
Response, %ΔHRSD	−0.46 (0.20)	−0.42 (0.24)	0.08	163	(38.72)	−0.65	0.11	258	(61.28)	−0.29	0.17	**<2.2 × 10** ^**−16**^
Stem-depressed, item-wise			0.07	373	(88.60)			48	(11.40)			**<2.2 × 10** ^**−16**^
not depressed	12 (2.85)	18 (4.27)		30	(7.13)			0	(0.00)			
mild	54 (12.83)	115 (27.32)		169	(40.14)			0	(0.00)			
moderate	47 (11.16)	127 (30.16)		174	(41.33)			0	(0.00)			
moderately severe	7 (1.66)	38 (9.03)		0	(0.00)			45	(10.69)			
severe	1 (0.24)	2 (0.48)		0	(0.00)			3	(0.71)			

^a^Analyses were based on 421 MDD subjects. ^b^Difference tests were conducted using Student *t*-test (for continuous data) or Chi-square test (for categorical data).

**Figure 1:**
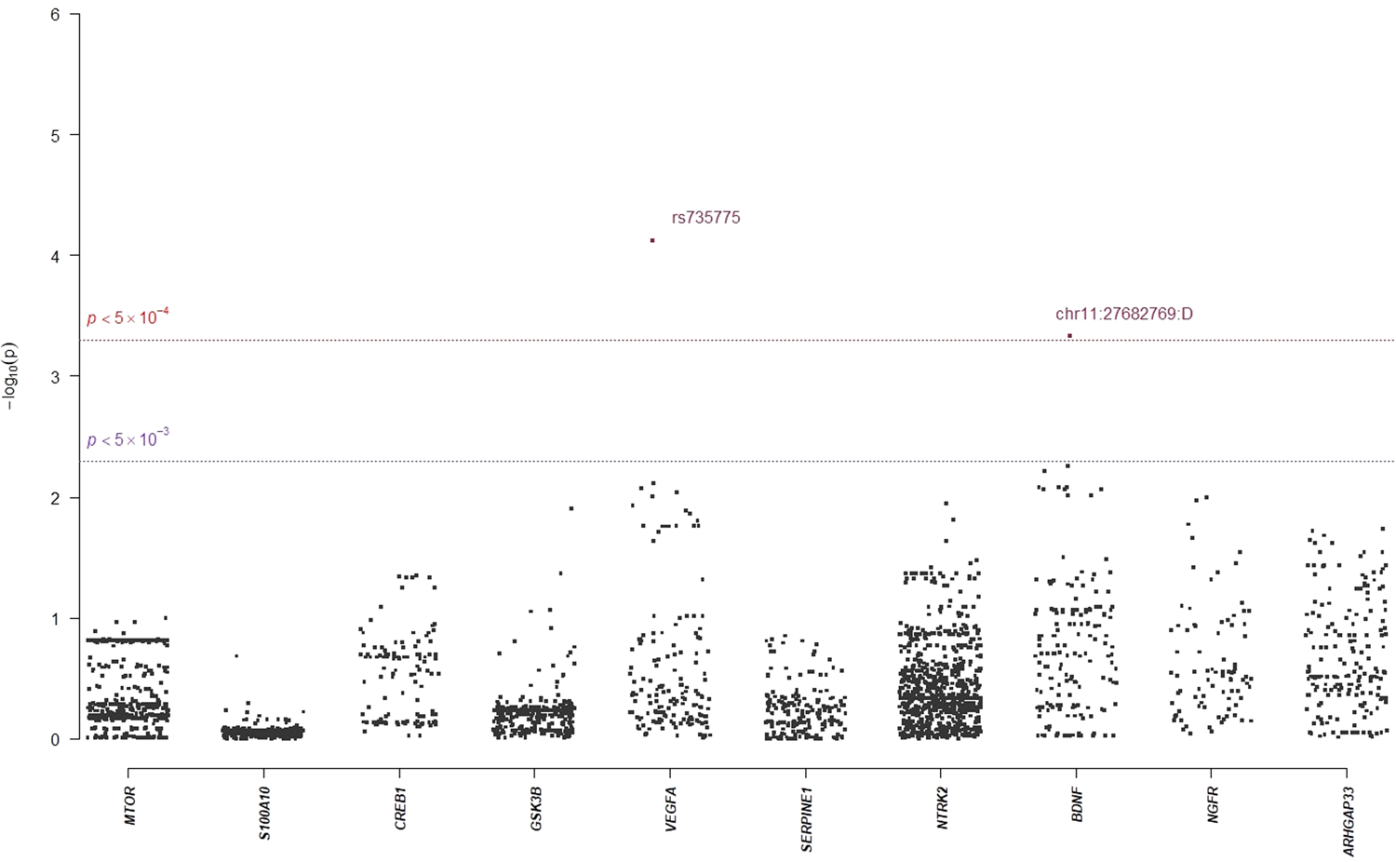
Manhattan plot of meta-analysis of selected candidate genes for Remitted (Score).

**Figure 2:**
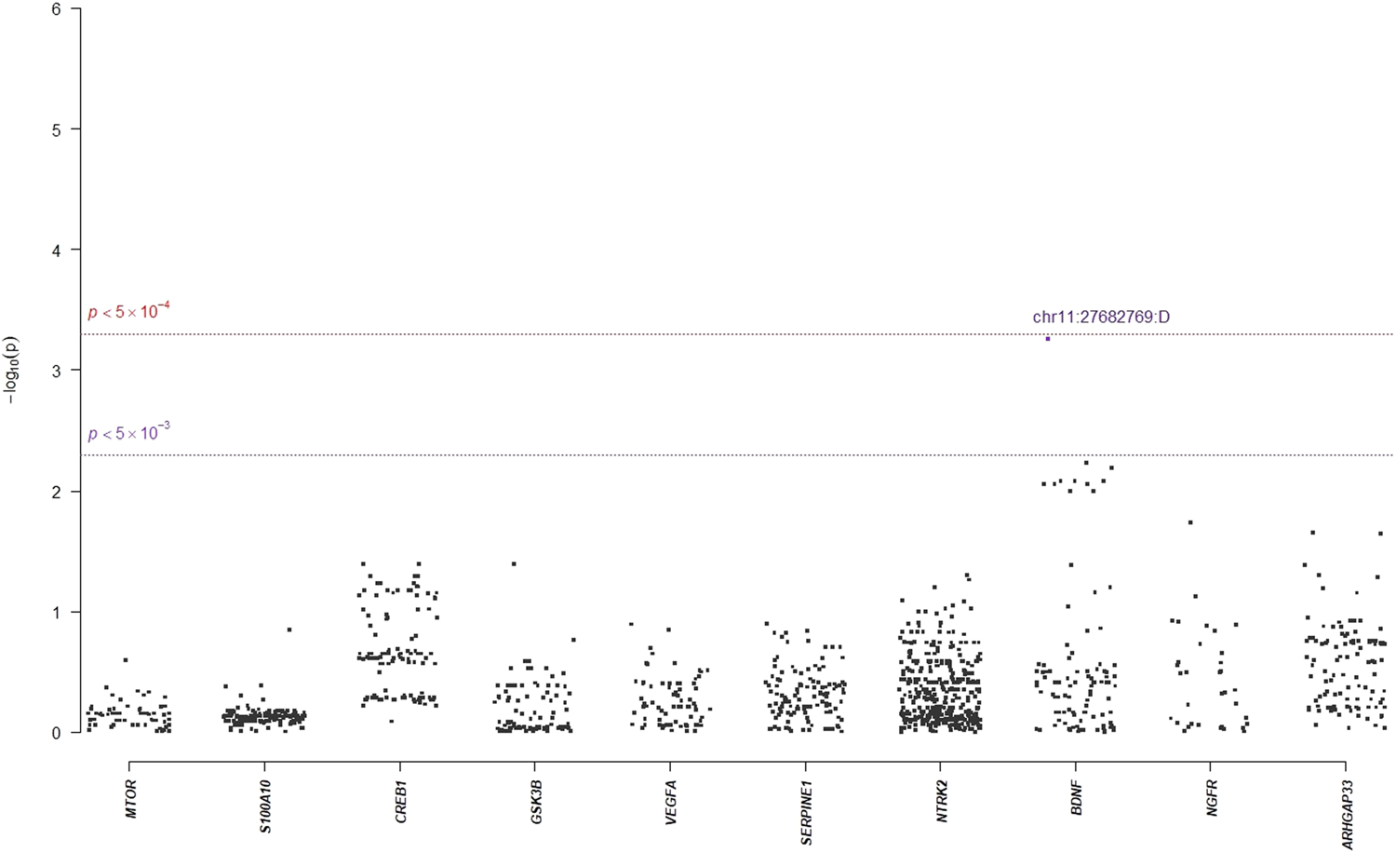
Manhattan plot of mega-analysis of selected candidate genes for Remitted (Score).

**Figure 3:**
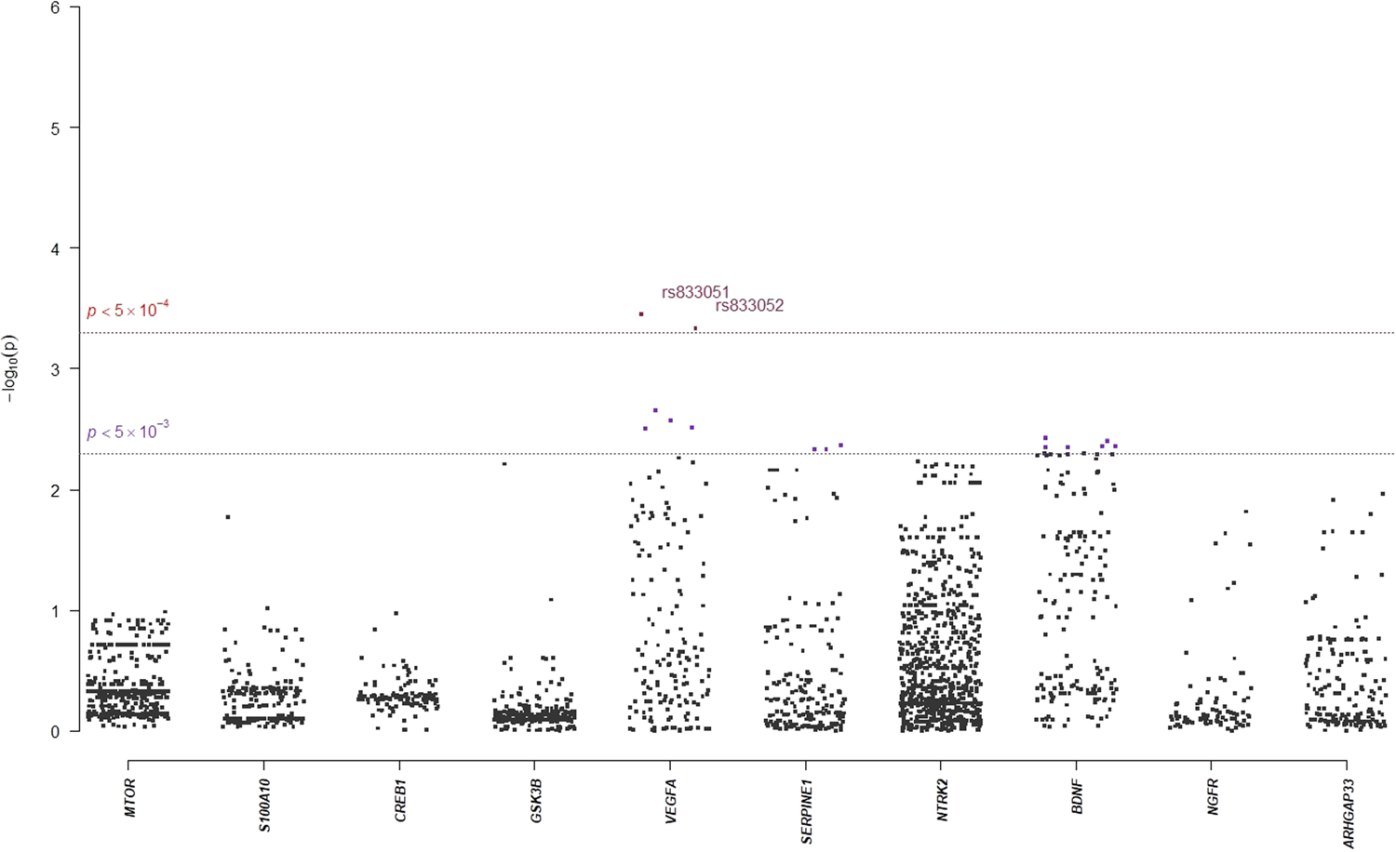
Manhattan plot of meta-analysis of selected candidate genes for Response (%ΔHRSD, continuous).

**Figure 4:**
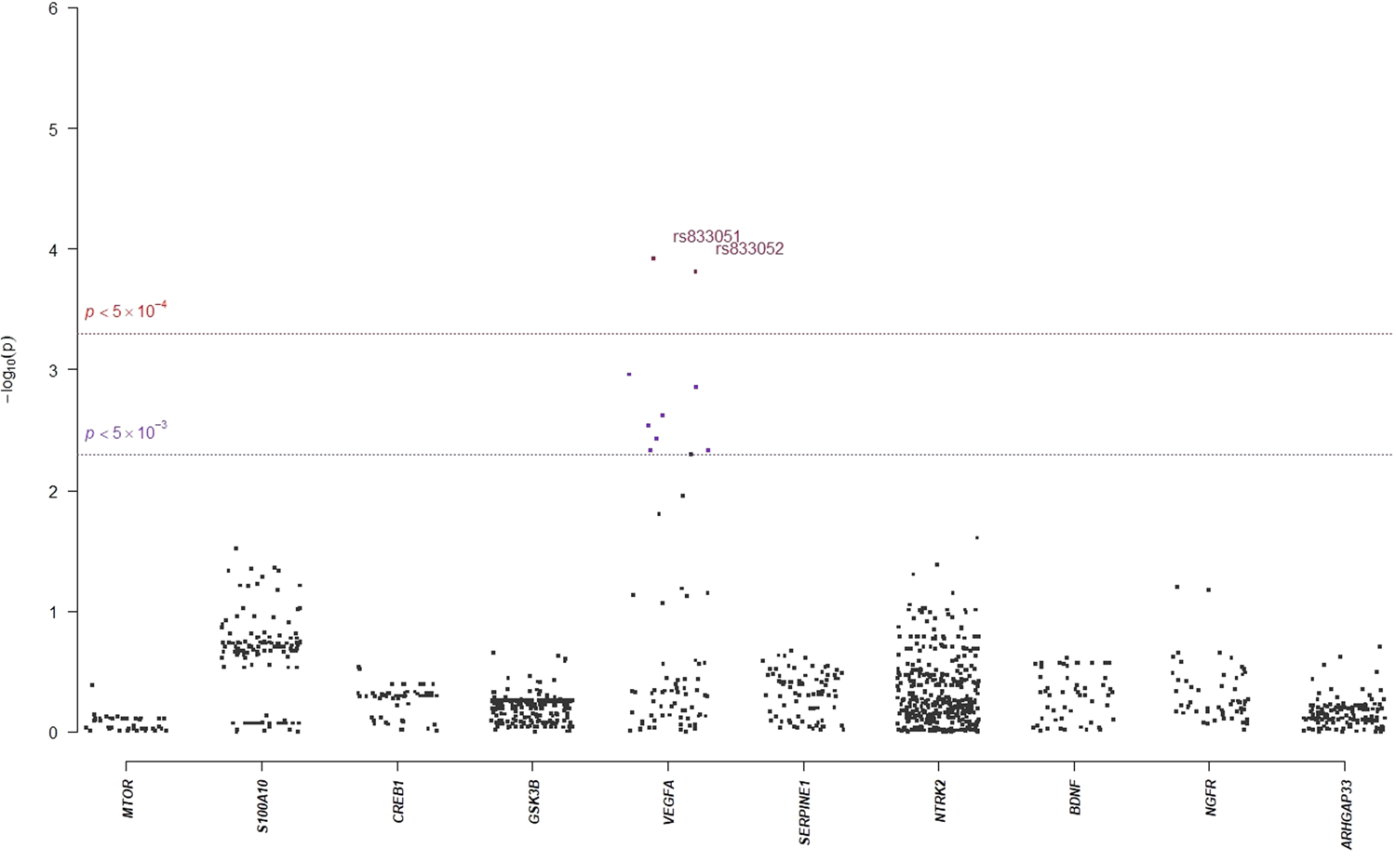
Manhattan plot of mega-analysis of selected candidate genes for Response (%ΔHRSD, continuous).

In single-marker association analyses, 64 SNPs with odds ratios (ORs) ranged from 0.18 to 2.88 in NHRI samples and ranged from 0.19 to 4.22 in TVGH samples (0.92–1.99 in combined samples) were reported to have suggestive signals with treatment-response phenotypes, which reached *p*-value < 5.0 × 10^−3^ either in mega-analysis or in meta-analysis (Table 3). Among them, only six SNPs reached significant levels (*p*-value < 5.0 × 10^−3^) in both meta-analysis and mega-analysis. One SNP, chr11:27682769:D (mapped to *BDNF*) was significantly associated with remitted (Score) (please see Figs 1 and 2 for their Manhattan plot of meta-analysis and mega-analysis), and 5 SNPs (rs6920449, rs1535507, rs833051, rs833052, and rs2148252), which were mapped to *VEGFA*, were significantly associated with response (%ΔHRSD, continuous) (please see Figs 3 and 4 for their Manhattan plot of meta-analysis and mega-analysis). Other 58 SNPs were significantly associated with treatment-response phenotypes in either meta-analysis or mega-analysis. Results of gene-based association analyses are listed in Table 4. Only *BDNF* gene in remitted (Score) and *VEGFA* gene in response (%ΔHRSD, binary and continuous) showed suggestive signals (*p*-values < 5.0 × 10^−2^) in mega-analysis and/or meta-analysis. These indicated that the possible mechanisms of association may be due to the neurotrophic pathway and serotonin system pathway. Furthermore, the most studied polymorphism Val66Met (rs6265) in *BDNF* is included in particular and the results of single-marker association and gene-based association were non-suggestive.

**Table 3:** *BDNF*-related susceptibility loci for major depressive disorder that related to response to antidepressant drug treatment in Han Chinese.

SNP	CHR: Position	Mapped gene	NHRI			TVGM			Mega-analysis			Meta-analysis *p*-value
			MAF	OR/$\hat{\beta }$	*p*-value	MAF	OR/$\hat{\beta }$	*p*-value	MAF	OR/$\hat{\beta }$	*p*-value	
**Remitted (Score, binary)**												
rs735775	6:43705011	*VEGFA*	0.2411	0.72	1.3 × 10^−1^	0.2411	4.22	4.2 × 10^−5^	0.2411	1.16	3.9 × 10^−1^	**7.6 × 10** ^**−5**^
chr11:27682769:D	11:27682769	*BDNF*	0.1397	2.11	4.9 × 10^−3^	0.1677	2.45	8.5 × 10^−3^	0.1495	1.99	**5.6 × 10** ^**−4**^	**4.7 × 10** ^**−4**^
**Response (%ΔHRSD, binary)**												
rs25648	6:43738977	*VEGFA*	0.0553	0.32	2.7 × 10^−2^	0.0685	3.07	1.9 × 10^−2^	0.0596	0.99	9.6 × 10^−1^	**4.4 × 10** ^**−3**^
rs7044702	9:87289051	*NTRK2*	0.0425	0.29	4.5 × 10^−2^	0.0804	3.29	8.2 × 10^−3^	0.0570	1.19	5.5 × 10^−1^	**3.3 × 10** ^**−3**^
rs72737649	9:87290163		0.0427	0.29	4.7 × 10^−2^	0.0804	3.29	8.2 × 10^−3^	0.0571	1.20	5.4 × 10^−1^	**3.4 × 10** ^**−3**^
rs12555206	9:87291075		0.0427	0.29	4.7 × 10^−2^	0.0804	3.29	8.2 × 10^−3^	0.0571	1.20	5.4 × 10^−1^	**3.4 × 10** ^**−3**^
rs56165347	9:87291653		0.0427	0.29	4.7 × 10^−2^	0.0804	3.29	8.2 × 10^−3^	0.0571	1.20	5.4 × 10^−1^	**3.4 × 10** ^**−3**^
rs72737651	9:87292681		0.0427	0.29	4.7 × 10^−2^	0.0778	3.63	5.5 × 10^−3^	0.0561	1.23	4.7 × 10^−1^	**2.4 × 10** ^**−3**^
rs72737652	9:87293026		0.0427	0.29	4.7 × 10^−2^	0.0804	3.29	8.2 × 10^−3^	0.0571	1.20	5.4 × 10^−1^	**3.4 × 10** ^**−3**^
rs78521596	9:87293376		0.0427	0.29	4.7 × 10^−2^	0.0804	3.29	8.2 × 10^−3^	0.0571	1.20	5.4 × 10^−1^	**3.4 × 10** ^**−3**^
rs12555252	9:87294474		0.0386	0.18	2.7 × 10^−2^	0.0719	3.31	1.2 × 10^−2^	0.0513	1.13	7.0 × 10^−1^	**2.9 × 10** ^**−3**^
rs56134605	9:87295392		0.0386	0.18	2.7 × 10^−2^	0.0719	3.31	1.2 × 10^−2^	0.0513	1.13	7.0 × 10^−1^	**2.9 × 10** ^**−3**^
rs72737657	9:87296981		0.0386	0.18	2.7 × 10^−2^	0.0719	3.31	1.2 × 10^−2^	0.0513	1.13	7.0 × 10^−1^	**2.9 × 10** ^**−3**^
rs12554121	9:87297194		0.0386	0.18	2.7 × 10^−2^	0.0719	3.31	1.2 × 10^−2^	0.0513	1.13	7.0 × 10^−1^	**2.9 × 10** ^**−3**^
rs12554152	9:87297361		0.0385	0.18	2.6 × 10^−2^	0.0719	3.31	1.2 × 10^−2^	0.0512	1.12	7.2 × 10^−1^	**2.8 × 10** ^**−3**^
rs72737659	9:87301740		0.0435	0.34	6.0 × 10^−2^	0.0744	3.55	7.2 × 10^−3^	0.0549	1.25	4.6 × 10^−1^	**3.8 × 10** ^**−3**^
chr9:87301843:D	9:87301843		0.0435	0.34	6.0 × 10^−2^	0.0744	3.55	7.2 × 10^−3^	0.0549	1.25	4.6 × 10^−1^	**3.8 × 10** ^**−3**^
rs72737661	9:87303166		0.0435	0.34	6.0 × 10^−2^	0.0744	3.55	7.2 × 10^−3^	0.0549	1.25	4.6 × 10^−1^	**3.8 × 10** ^**−3**^
rs74999771	9:87304265		0.0435	0.34	6.0 × 10^−2^	0.0744	3.55	7.2 × 10^−3^	0.0549	1.25	4.6 × 10^−1^	**3.8 × 10** ^**−3**^
rs142208864	9:87305126		0.0435	0.34	6.0 × 10^−2^	0.0744	3.55	7.2 × 10^−3^	0.0549	1.25	4.6 × 10^−1^	**3.8 × 10** ^**−3**^
rs113419923	9:87311079		0.0435	0.34	6.0 × 10^−2^	0.0744	3.55	7.2 × 10^−3^	0.0549	1.25	4.6 × 10^−1^	**3.8 × 10** ^**−3**^
rs111789878	9:87313093		0.0435	0.34	6.0 × 10^−2^	0.0744	3.55	7.2 × 10^−3^	0.0549	1.25	4.6 × 10^−1^	**3.8 × 10** ^**−3**^
rs112534036	9:87313387		0.0417	0.26	3.5 × 10^−2^	0.0744	3.55	7.2 × 10^−3^	0.0539	1.19	5.6 × 10^−1^	**2.4 × 10** ^**−3**^
rs3780629	9:87316608		0.0435	0.34	6.0 × 10^−2^	0.0744	3.55	7.2 × 10^−3^	0.0549	1.25	4.6 × 10^−1^	**3.8 × 10** ^**−3**^
rs112182030	9:87317690		0.0435	0.34	6.0 × 10^−2^	0.0744	3.55	7.2 × 10^−3^	0.0549	1.25	4.6 × 10^−1^	**3.8 × 10** ^**−3**^
rs143407628	9:87319345		0.0435	0.34	6.0 × 10^−2^	0.0744	3.55	7.2 × 10^−3^	0.0549	1.25	4.6 × 10^−1^	**3.8 × 10** ^**−3**^
rs7020467	9:87331404		0.0435	0.34	6.0 × 10^−2^	0.0744	3.55	7.2 × 10^−3^	0.0549	1.25	4.6 × 10^−1^	**3.8 × 10** ^**−3**^
chr9:87436260:D	9:87436260		0.2996	0.75	1.6 × 10^−1^	0.3720	2.08	3.6 × 10^−3^	0.3254	1.11	5.0 × 10^−2^	**4.8 × 10** ^**−3**^
**Response (%ΔHRSD, continuous)**												
rs6920449	6:43710348	*VEGFA*	0.2689	0.08	1.7 × 10^−3^	0.3155	0.03	1.7 × 10^−1^	0.2864	0.06	**1.1 × 10** ^**−3**^	**3.6 × 10** ^**−3**^
rs945128	6:43710756		0.3281	0.07	6.2 × 10^−3^	0.3661	0.03	1.8 × 10^−1^	0.3411	0.05	**4.7 × 10** ^**−3**^	8.9 × 10^−3^
rs945129	6:43710959		0.3281	0.07	6.2 × 10^−3^	0.3661	0.03	1.8 × 10^−1^	0.3411	0.05	**4.7 × 10** ^**−3**^	8.9 × 10^−3^
rs4714695	6:43711459		0.3261	0.07	4.7 × 10^−3^	0.3661	0.03	1.9 × 10^−1^	0.3400	0.05	**3.8 × 10** ^**−3**^	7.2 × 10^−3^
rs1535506	6:43711847		0.3281	0.07	6.2 × 10^−3^	0.3653	0.03	1.8 × 10^−1^	0.3407	0.05	**5.0 × 10** ^**−3**^	9.6 × 10^−3^
rs1535507	6:43711981		0.2688	0.08	1.6 × 10^−3^	0.3125	0.03	2.0 × 10^−1^	0.2850	0.06	**1.4 × 10** ^**−3**^	**3.6 × 10** ^**−3**^
rs7747520	6:43712351		0.3280	0.07	7.2 × 10^−3^	0.3636	0.04	9.1 × 10^−2^	0.3400	0.05	**2.9 × 10** ^**−3**^	5.5 × 10^−3^
rs833051	6:43722953		0.2794	0.09	6.3 × 10^−4^	0.3263	0.04	5.0 × 10^−2^	0.2969	0.07	**1.2 × 10** ^**−4**^	**7.7 × 10** ^**−4**^
rs833052	6:43723335		0.2747	0.08	8.7 × 10^−4^	0.3274	0.05	4.8 × 10^−2^	0.2944	0.06	**1.5 × 10** ^**−4**^	**4.7 × 10** ^**−4**^
rs866236	6:43726956		0.4723	0.07	4.6 × 10^−3^	0.4970	0.01	5.2 × 10^−1^	0.4813	0.04	1.1 × 10^−2^	**1.1 × 10** ^**−3**^
rs3025035	6:43751359		0.1383	0.07	3.5 × 10^−2^	0.1429	0.07	2.1 × 10^−2^	0.1449	0.07	**2.4 × 10** ^**−3**^	6.0 × 10^−3^
rs2148252	6:43794144		0.3182	−0.04	9.1 × 10^−2^	0.2798	−0.07	3.8 × 10^−3^	0.3014	−0.05	**3.2 × 10** ^**−3**^	**3.1 × 10** ^**−3**^
rs2410797	7:100818591	*SERPINE1*	0.0652	−0.12	4.9 × 10^−3^	0.0719	−0.07	1.1 × 10^−1^	0.0691	−0.10	1.7 × 10^−1^	**4.6 × 10** ^**−3**^
rs10236771	7:100819529		0.0652	−0.12	4.9 × 10^−3^	0.0719	−0.07	1.1 × 10^−1^	0.0691	−0.10	1.7 × 10^−1^	**4.6 × 10** ^**−3**^
rs10233391	7:100822335		0.0657	−0.13	4.5 × 10^−3^	0.0714	−0.07	1.1 × 10^−1^	0.0693	−0.10	1.6 × 10^−1^	**4.3 × 10** ^**−3**^
rs10767662	11:27705615	*BDNF*	0.3980	0.03	2.2 × 10^−1^	0.3789	−0.07	2.4 × 10^−3^	0.3898	−0.01	5.1 × 10^−1^	**4.4 × 10** ^**−3**^
rs4542361	11:27707681		0.4113	0.03	1.4 × 10^−1^	0.3939	−0.07	3.4 × 10^−3^	0.4036	−0.01	7.2 × 10^−1^	**4.0 × 10** ^**−3**^
rs4385847	11:27707729		0.4120	0.04	1.3 × 10^−1^	0.3939	−0.07	3.4 × 10^−3^	0.4040	−0.01	7.2 × 10^−1^	**3.8 × 10** ^**−3**^
rs4378341	11:27707831		0.4000	0.03	2.2 × 10^−1^	0.3789	−0.07	2.4 × 10^−3^	0.3910	−0.01	5.0 × 10^−1^	**4.5 × 10** ^**−3**^
rs7482752	11:27708242		0.3926	0.03	2.1 × 10^−1^	0.3781	−0.07	2.4 × 10^−3^	0.3863	−0.01	5.1 × 10^−1^	**4.4 × 10** ^**−3**^
rs2883187	11:27741092		0.4140	0.03	1.5 × 10^−1^	0.3946	−0.07	3.5 × 10^−3^	0.4040	−0.01	6.8 × 10^−1^	**4.5 × 10** ^**−3**^
**Stem-depressed (Item-wise)**												
rs17087481	9:87247045	*NTRK2*	0.1964	2.35	4.7 × 10^−2^	0.2078	0.23	5.2 × 10^−3^	0.2000	0.97	9.3 × 10^−1^	**2.3 × 10** ^**−3**^
rs72737624	9:87252225		0.1964	2.35	4.7 × 10^−2^	0.2104	0.23	5.9 × 10^−3^	0.2009	0.98	9.4 × 10^−1^	**2.5 × 10** ^**−3**^
rs1936433	9:87255047		0.1964	2.35	4.7 × 10^−2^	0.2104	0.23	5.9 × 10^−3^	0.2009	0.98	9.4 × 10^−1^	**2.5 × 10** ^**−3**^
rs72737626	9:87256613		0.1805	2.46	5.2 × 10^−2^	0.2013	0.19	3.2 × 10^−3^	0.1880	0.92	7.8 × 10^−1^	**1.6 × 10** ^**−3**^
rs72737629	9:87258004		0.1890	2.61	3.7 × 10^−2^	0.2013	0.19	3.2 × 10^−3^	0.1930	0.97	9.1 × 10^−1^	**1.2 × 10** ^**−3**^
rs1582245	9:87261279		0.1890	2.61	3.7 × 10^−2^	0.2013	0.19	3.2 × 10^−3^	0.1930	0.97	9.1 × 10^−1^	**1.2 × 10** ^**−3**^
rs1347859	9:87264718		0.1890	2.61	3.7 × 10^−2^	0.2013	0.19	3.2 × 10^−3^	0.1930	0.97	9.1 × 10^−1^	**1.2 × 10** ^**−3**^
rs11140720	9:87272824		0.1937	2.83	2.3 × 10^−2^	0.2113	0.23	5.8 × 10^−3^	0.1998	1.07	8.3 × 10^−1^	**1.3 × 10** ^**−3**^
rs11140721	9:87274748		0.1937	2.83	2.3 × 10^−2^	0.2113	0.23	5.8 × 10^−3^	0.1998	1.07	8.3 × 10^−1^	**1.3 × 10** ^**−3**^
rs1147199	9:87275895		0.1937	2.83	2.3 × 10^−2^	0.2113	0.23	5.8 × 10^−3^	0.1998	1.07	8.3 × 10^−1^	**1.3 × 10** ^**−3**^
chr9:87279570:I	9:87279570		0.2020	2.88	2.1 × 10^−2^	0.2261	0.25	1.1 × 10^−2^	0.2103	1.11	7.3 × 10^−1^	**2.2 × 10** ^**−3**^
rs1187321	9:87283031		0.1937	2.83	2.3 × 10^−2^	0.2113	0.23	5.8 × 10^−3^	0.1998	1.07	8.3 × 10^−1^	**1.3 × 10** ^**−3**^
rs1187326	9:87285915		0.2174	2.01	7.5 × 10^−2^	0.2351	0.27	6.3 × 10^−3^	0.2231	0.93	7.9 × 10^−1^	**4.1 × 10** ^**−3**^
rs1211166	9:87285992		0.1937	2.83	2.3 × 10^−2^	0.2113	0.23	5.8 × 10^−3^	0.1998	1.07	8.3 × 10^−1^	**1.3 × 10** ^**−3**^
rs56568126	9:87289257		0.1968	2.80	2.5 × 10^−2^	0.2096	0.26	1.1 × 10^−2^	0.2000	1.10	7.5 × 10^−1^	**2.5 × 10** ^**−3**^

Abbreviation: SNP, single nucleotide polymorphism; CHR, chromosome; MAF, minor allele frequency; OR, odds ratio.

^a^Association analyses were based on 421 MDD subjects, using linear (or logistic) regression with an additive model (1-degree of freedom) after adjusting for gender and age. ^b^Meta-analysis *p*-value was calculated using the Fisher exact method. ^c^Only SNPs that reached *p*-value at the level of 5.0 × 10^−4^ (or 5.0 × 10^−3^) either in mega-analysis or in meta-analysis were reported as significant (or suggestive) markers. ^d^SNPs highlighted in bold are chip markers.

**Table 4:** *BDNF*-related susceptibility genes for major depressive disorder that related to response to antidepressant drug treatment in Han Chinese

Gene	Pathway related to antidepressant effect	NHRI		TVGM		Mega-analysis		Meta-analysis *p*-value
		Prop.	*p*-value	Prop.	*p*-value	Prop.	*p*-value	
**Remitted (Score, binary)**								
*BDNF*	Neurotrophic/BDNF pathway	19/166	4.2×10^-2^	42/166	4.7×10^-2^	13/166	**8.4×10** ^**-3**^	**1.4×10** ^**-2**^
**Response (%ΔHRSD, binary)**								
*VEGFA*	Neurotrophic/BDNF pathway	31/156	5.5×10^-2^	21/156	2.8×10^-2^	16/156	2.8×10^-1^	**1.1×10** ^**-2**^
**Response (%ΔHRSD, continuous)**								
*VEGFA*	Neurotrophic/BDNF pathway	36/156	3.0×10^-2^	17/156	9.9×10^-2^	12/156	**5.0×10** ^**-2**^	**2.0×10** ^**-2**^
**Stem-depressed (Item-wise)**								
N/A								

Abbreviation: Prop, proportion of significant SNPs.

^a^Gene-based association analyses were based on 421 major depressive disorder patients. ^b^Only genes that reached *p*-value at the level of 0.01 (or 0.05) either in mega-analysis or in meta-analysis were reported as significant (or suggestive) loci, based on 50,000 permutations. ^c^Models were adjusted for gender and age. ^d^Proportion of significant SNPs was defined as the number of significant SNPs (*p*-value < 0.05) divided by total number of SNPs in that gene. ^e^We conducted linear (if continuous) and logistic (if dichotomous) regressions analyses for treatment-response phenotypes.

Table 5 exhibits summary of eQTL prediction. We found that 4 SNPs (rs1535507, rs833051, rs833052, rs2148252) exhibited direct eQTL effects (in total 9 hits). The rs1535507 SNP is involved in regulating expressions of *RSPH9* and *MAD2L1BP |VEGF* in dendritic cells and in blood tissues. The rs833051 SNP is involved in regulating expressions of *RP1-261G23.7* in cells transformed fibroblasts. The rs833052 SNP is involved in regulating expressions of *MAD2L1BP* and *MRPS18A* in blood tissue and in dendritic cells. The rs2148252 SNP is involved in regulating expressions of *VEGFA* in dendritic cells. The other two SNPs (rs6920449 and chr11:27682769:D) do not act as eQTLs, but SNPs in high LD (r2 ≥ 0.8) with rs6920449 are predicted to be eQTLs, which regulated several gene expressions, including *MAD2L1BP* (blood tissue), *VEGFA* (blood tissue), and *RSPH9* (dendritic cells).

**Table 5:** Summary of eQTL prediction.

Chr	Position	LD (r^2^)	LD (D’)	Variant	Ref	Alt	EUR freq	Enhancer histone marks	DNAse	Proteins bound	Motifs changed	GRASP QTL hits	Selected eQTL hits	GENCODE genes
**(a) Query SNPs: rs6920449, rs1535507 and variants with r**^**2**^** ≥ 0.8 and eQTL hits**.														
6	43729450	0.83	0.96	rs9472115	G	A	0.62	SKIN, GI			Homez		3 hits	RP1-261G23.5
6	43740785	1	1	rs7764284	A	T	0.67	16 tissues		ZNF263	GR, HNF4, NRSF			3 kb 5′ of RP1-261G23.5
6	43742611	1	1	**rs6920449**	T	C	0.67	8 tissues	IPSC		4 altered motifs			4.8 kb 5′ of RP1-261G23.5
6	43744244	1	1	**rs1535507**	T	C	0.67	10 tissues		FOXA1, FOXA2	NF-kappaB, ZBTB7A	1 hit	3 hits	6.4 kb 5′ of RP1-261G23.5
**(b) Query SNPs: rs833051 and variants with r**^**2**^** ≥ 0.8 and eQTL hits**.														
6	43755216	1	1	**rs833051**	A	T	0.65				SRF		1 hit	15 kb 5′ of VEGFA
6	43755598	0.99	1	rs833052	A	C	0.65					2 hits	1 hit	15 kb 5′ of VEGFA
**(C) Query SNPs: rs833052 and variants with r**^**2**^** ≥ 0.8 and eQTL hits**.														
6	43755216	0.99	1	rs833051	A	T	0.65				SRF		1 hit	15 kb 5′ of VEGFA
6	43755598	1	1	**rs833052**	A	C	0.65					2 hits	1 hit	15 kb 5′ of VEGFA
**(d) Query SNPs: rs2148252 and variants with r**^**2**^** > = 0.8 and variants with r**^**2**^** ≥ 0.8 and eQTL hits**.														
6	43826407	1	1	**rs2148252**	G	A	0.34	9 tissues			4 altered motifs	1 hit		25 kb 3′ of RP11-344J7.2

We also conducted a case-control association study using samples from NHRI and TVGH (421 MDD patients) and Taiwan Biobank (2,998 healthy controls). We found that all reported SNPs and genes did not reach the suggestive association level.

## Discussion

The major finding is that 64 SNPs were reported to have suggestive signals with treatment-response phenotypes either in mega-analysis or in meta-analysis. Among them, 6 SNPs reached a significant level (*p*-value < 5.0 × 10^−3^) in both meta-analysis and mega-analysis. The genetic association directions of the 6 SNPs are the same in both study groups. The other 58 SNPs were significantly associated with treatment-response phenotypes in either meta-analysis or mega-analysis. Further, among the 10 genes tested, *BDNF* gene (in remitted) and *VEGFA* gene (in response) were suggestive markers for treatment-response phenotypes using samples from NHRI, TVGH, and the combined.

BDNF is essential for the mechanism of pharmacological therapies currently used to treat MDD^39,40^. Both *BDNF* mRNA and protein expressions are increased after long-term medication with antidepressants, including SSRIs, in the hippocampus in animal models^9,41^. Moreover, infusion of BDNF into the dentate gyrus of hippocampus produced antidepressant-like behaviors^42^. In support of these findings, a recent study demonstrated that knockdown of *TrkB*, but not *Bdnf*, in the dorsal raphe nucleus of mice results in loss of antidepressant efficacy^43^. Recent meta-analyses showed strong evidence that peripheral BDNF levels were lower in MDD subjects than healthy control subjects, and BDNF levels significantly increased after antidepressant treatment^12,41^. From the above findings, *BDNF* has been tested in many antidepressant pharmacogenetic studies, especially the functional *BDNF* Val66Met polymorphism^16,44^. However, most studies showed no association between this polymorphism and antidepressant response^16^. Meta-analysis of seven studies and the STAR*D data including a total of 3,128 subjects showed that the *BDNF* Val66Met polymorphism was not significantly associated with antidepressant therapeutic response^45^. In this study we found no association between this *BDNF* Val66Met polymorphism and SSRI therapeutic response. However, we found one SNP, chr11:27682769:D (mapped to *BDNF*), to be significantly associated with the remitted (Score). This SNP had ORs that were in the same direction in NHRI samples and TVGH samples, and our results of meta-analysis and mega-analysis suggested a statistically significant association between this SNP and the remitted. There was no eQTL effect for this SNP. It is still not clear how the mechanism of the chr11:27682769:D SNP is related to the SSRI therapeutic mechanism. Further studies are required to explore the functions of this *BDNF* SNP.

The other 5 SNPs (rs6920449, rs1535507, rs833051, rs833052, and rs2148252), which were significantly associated with SSRI therapeutic response, were mapped to *VEGFA*. VEGF is a member of growth factor family and has been implicated in neurotrophy and neurogenesis, which plays a pivotal role in brain development^46^. An animal study found that antidepressant drugs were shown to induce hippocampal expression of VEGF and that VEGF signaling through the Flk-1 receptor is required for antidepressant-induced cell proliferation^47^. In addition, the same research group demonstrated that infusions of inhibitors of VEGF-Flk-1 receptor signaling blocks the antidepressant-like activity of fluoxetine^47^. Study involving 30 MDD patients found that plasma VEGF levels significantly increased its association with clinical response with antidepressant^48^. With the significant role of VEGF in antidepressant action, we have tested 7 *VEGFA* polymorphisms and SSRI antidepressant response in 351 MDD patients^49^. No significant association with SSRI antidepressant therapeutic effect was shown in the alleles and genotypes of single loci, or haplotypes constructed from these polymorphisms. In this study, we also found no significant association between the 5 *VEGFA* SNPs and SSRI antidepressant response in both SNP-based and haplotype-based (data not shown) analyses using binary outcome (i.e., dichotomized %ΔHRSD), which is similar to the 7 *VEGFA* polymorphisms reported in our previous study. However, the 5 *VEGFA* SNPs found to be associated with SSRI antidepressant response using continuous outcome (i.e., %ΔHRSD) were different from the 7 *VEGFA* polymorphisms reported in our previous study, suggesting its simplicity of dichotomizing continuous variables gained at some cost (i.e., some of the information lost) in pharmacogenetic research. One benefit of using high-density imputed SNP mapping of antidepressant candidate genes is that some potentially significant signals for antidepressant treatment response could not be missed in pharmacogenetic studies.

We used HaploReg to predict a possible functional role of these 5 *VEGFA* SNPs. We found that 4 SNPs (rs1535507, rs833051, rs833052, and rs2148252) exhibited direct eQTL effects. Of note, functional annotation by HaploReg indicated that transcriptional regulation activity exists at the rs1535507, rs833052, and rs2148252 loci for the *VEGFA* gene in blood tissue and/or dendritic cells. Their functional impacts in antidepressant mechanism still need to be elucidated by further experimental studies.

Neurotrophic factors comprise major regulatory mechanisms controlling normal brain plasticity and neurogenesis. Dysfunctions of these systems form the basis for development of MDD^50^. For case-control association study using samples from NHRI and TVGH (421 MDD patients) and HCCGB (2,998 healthy controls), all these reported SNPs and genes did not reach significant level. This suggested that these tested SNPs and genes related to the neurotrophic pathway were not associated with MDD susceptibility. MDD is a complex mental disorder with a partly genetic etiology and each risk genetic locus confers relatively small increments in risk^51^. The negative association finding could be due to our sample size which lacks the statistical power to detect common variants of MDD susceptibility.

The major limitation of this study is the lack of a placebo lead-in design. It is well known that the placebo response plays an important role in antidepressant therapeutic response^52^. The second limitation of this study is that some potential confounding factors, such as side effects, incompliance or drop-out, were not under consideration, which might disturb the analysis in detecting a difference between the two treatment response groups. The third limitation is that our inclusion criterion for HRSD minimum score is 14, which is lower than other studies. Finally, plasma levels of antidepressants were not analyzed. Poor antidepressant response may result from sub-therapeutic plasma drug concentrations^53^.

In conclusion, our gene-based association identifies loci in *BDNF* and *VEGFA* as potential genetic predictors for SSRI therapeutic response in MDD patients. Future studies are needed to delineate the precise mechanism by which these genetic variants may influence antidepressant therapeutic response.

## Electronic supplementary material

Supplementary figures
