# Supplementary material for: Gene-based analysis of genes related to neurotrophic pathway suggests association of BDNF and VEGFA with antidepressant treatment-response in depressed patients
Source: Sci Rep. 2018 May 3;8:6983. doi: 10.1038/s41598-018-25529-y (PMC5934385; doi:10.1038/s41598-018-25529-y)
Supplement: Supplementary file 1 — Supplementary figures [file 41598_2018_25529_MOESM1_ESM.pdf]

Gene-based analysis of genes related to neurotrophic pathway suggests association of *BDNF* and *VEGFA* with antidepressant treatment-response in depressed patients

Chung-Feng Kao[1], Yu-Li Liu[2], Younger W-Y Yu[3], Albert C. Yang[4,5,6,7], Eugene Lin[8,9], Po-Hsiu Kuo[10]* & Shih-Jen Tsai [4,5,7]*

[1] Department of Agronomy, National Chung Hsing University, Taichung, Taiwan

[2] Center for Neuropsychiatric Research, National Health Research Institutes, Miaoli County, Taiwan

[3] Yu's Psychiatric Clinic, Kaohsiung, Taiwan

[4] Department of Psychiatry, Taipei Veterans General Hospital, Taipei, Taiwan

[5] Division of Psychiatry, National Yang-Ming University, Taipei, Taiwan

[6] Division of Interdisciplinary Medicine and Biotechnology, Beth Israel Deaconess Medical Center/Harvard Medical School, Boston, MA 02215, USA

[7] Institute of Brain Science, National Yang-Ming University, Taipei, Taiwan

[8] Graduate Institute of Biomedical Sciences, China Medical University, Taichung, Taiwan

[9] Department of Electrical Engineering, University of Washington, Seattle, WA 98195, USA

[10] Department of Public Health, Institute of Epidemiology and Preventive Medicine, National Taiwan University, Taipei, Taiwan

*** Corresponding author:**

E-mail: phkuo@ntu.edu.tw (P.-H.K.)

or

E-mail: tsai610913@gmail.com (S.-J.T.)

Short Title: Gene-based analysis of antidepressant effect

Figure S1

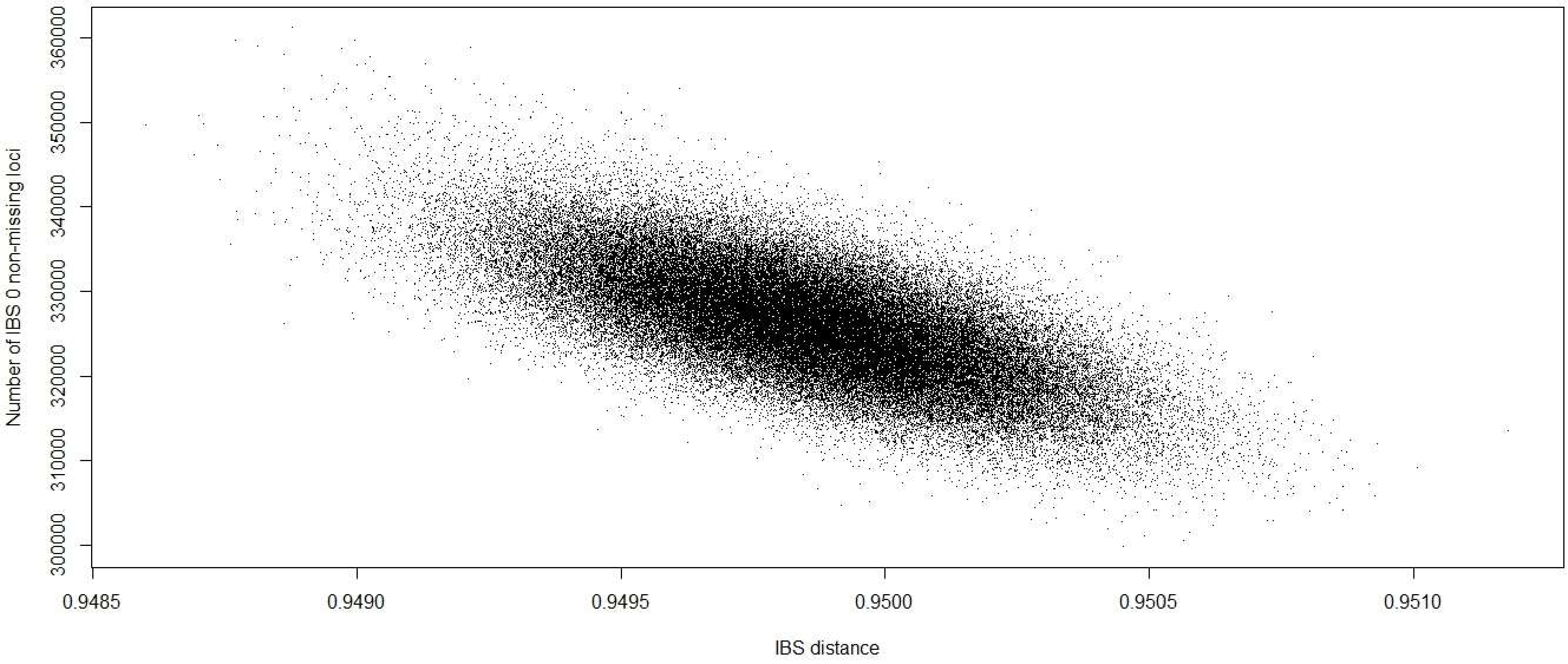

Number of IBS 0 non-missing loci
300000
310000
320000
330000
340000
350000
360000
0.9485
0.9490
0.9495
0.9500
0.9505
0.9510
IBS distance

Figure S2

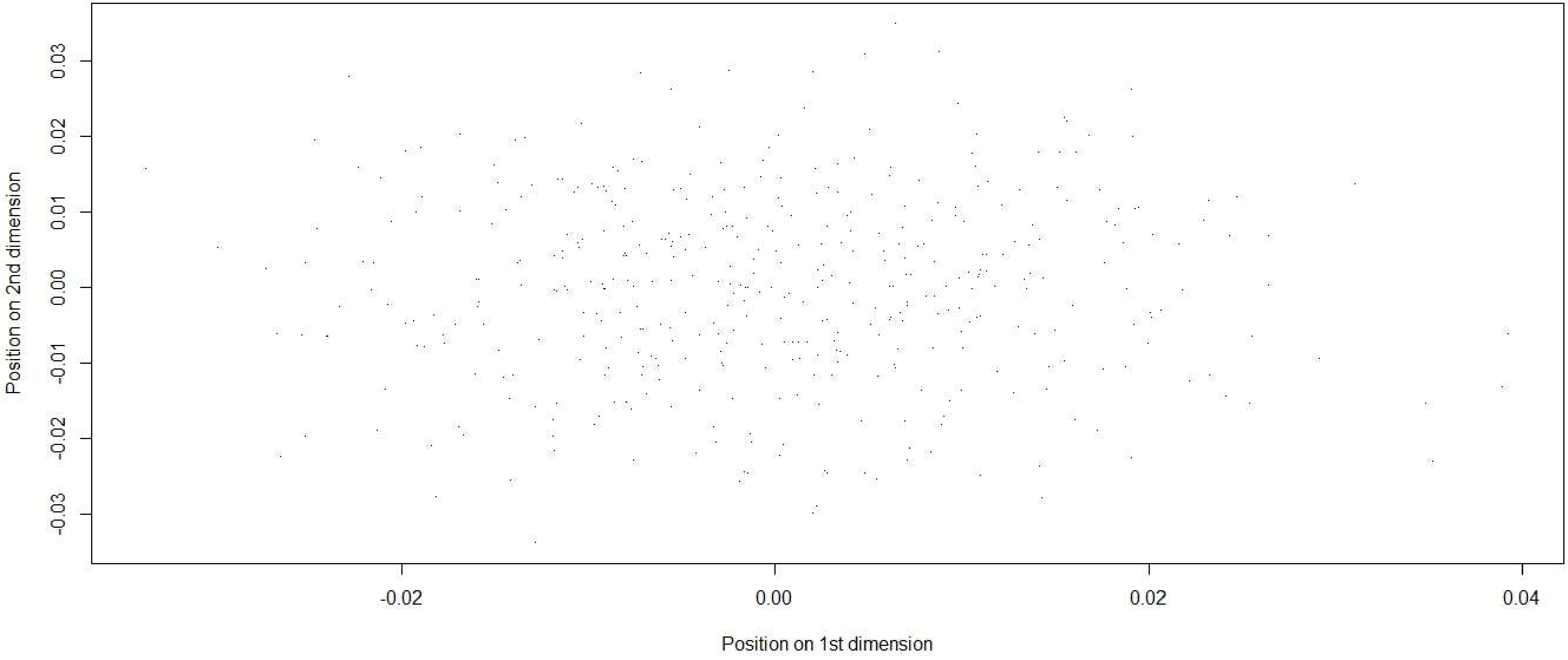

0.03
0.02
0.01
0.00
-0.01
-0.02
-0.03
Position on 2nd dimension
-0.02
0.00
0.02
0.04
Position on 1st dimension

Figure S3

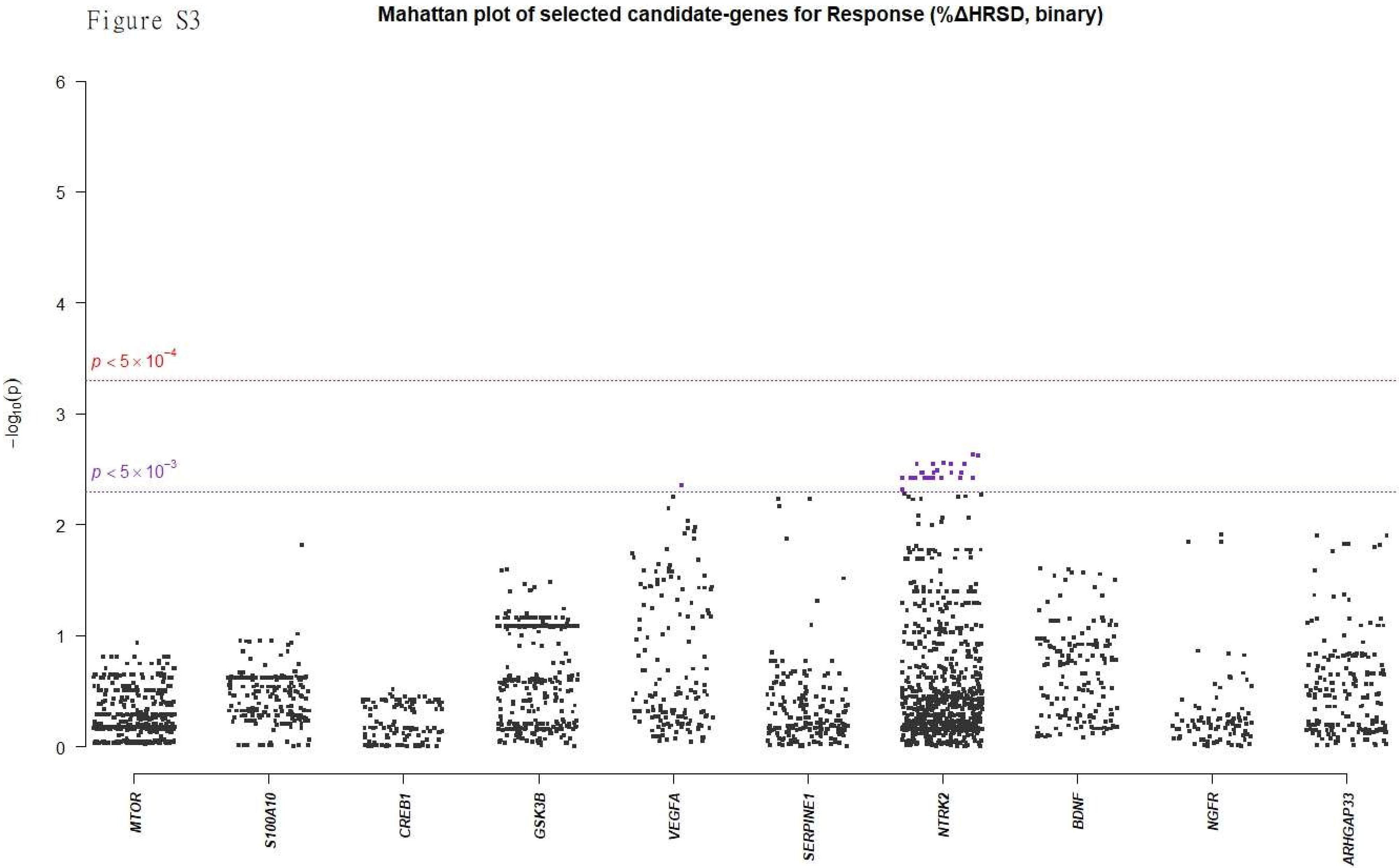
Mahattan plot of selected candidate-genes for Response (%ΔHRSD, binary)
6
5
4
3
2
1
0
−log10(p)
p < 5 × 10−4
p < 5 × 10−3
MTOR
S100A10
CREB1
GSK3B
VEGFA
SERPINE1
NTRK2
BDNF
NGFR
ARHGAP33

Figure S4

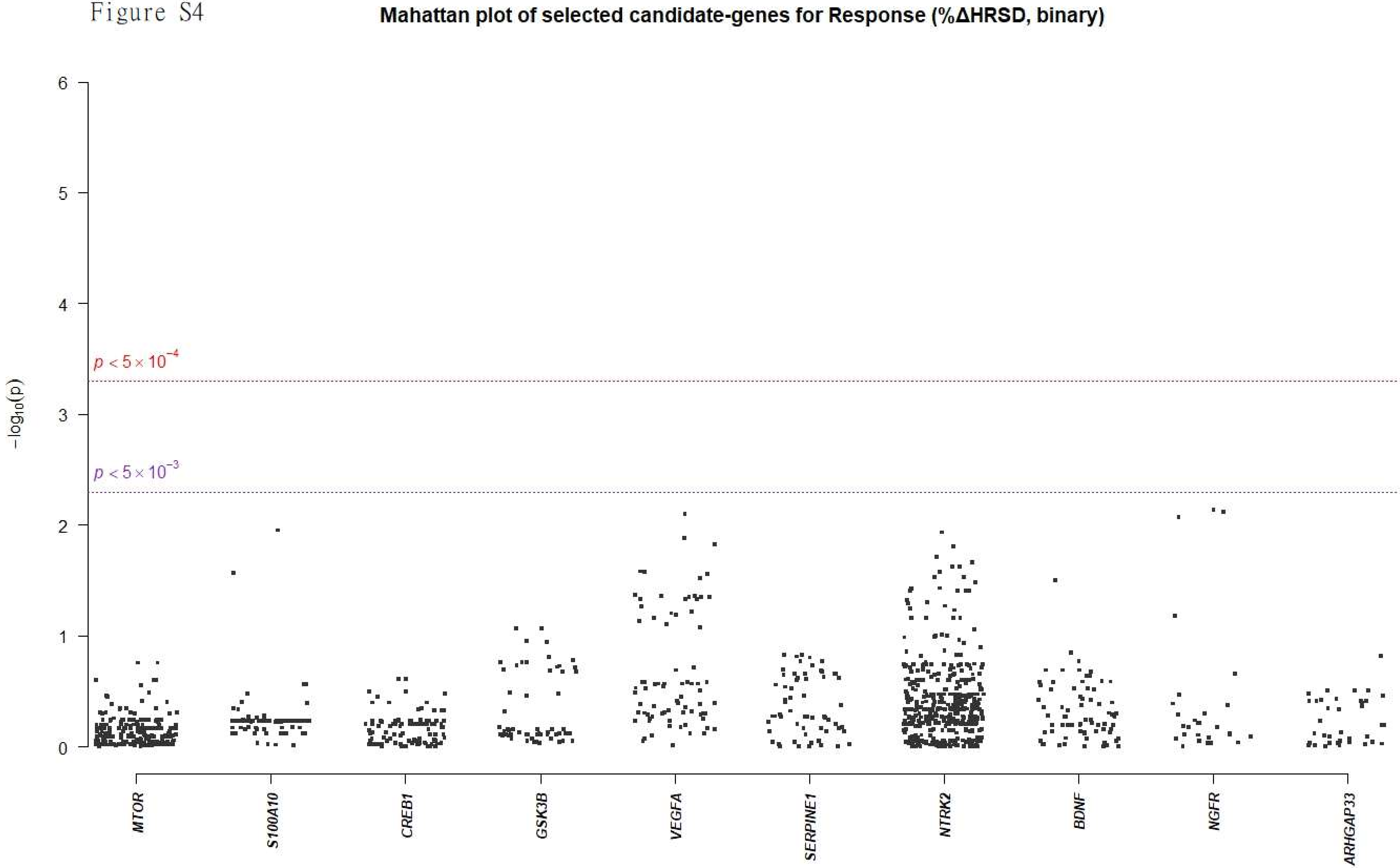
Mahattan plot of selected candidate-genes for Response (%ΔHRSD, binary)
6
5
4
3
2
1
0
−log10(p)
p < 5 × 10−4
p < 5 × 10−3
MTOR
S100A10
CREB1
GSK3B
VEGFA
SERPINE1
NTRK2
BDNF
NGFR
ARHGAP33

Figure S5

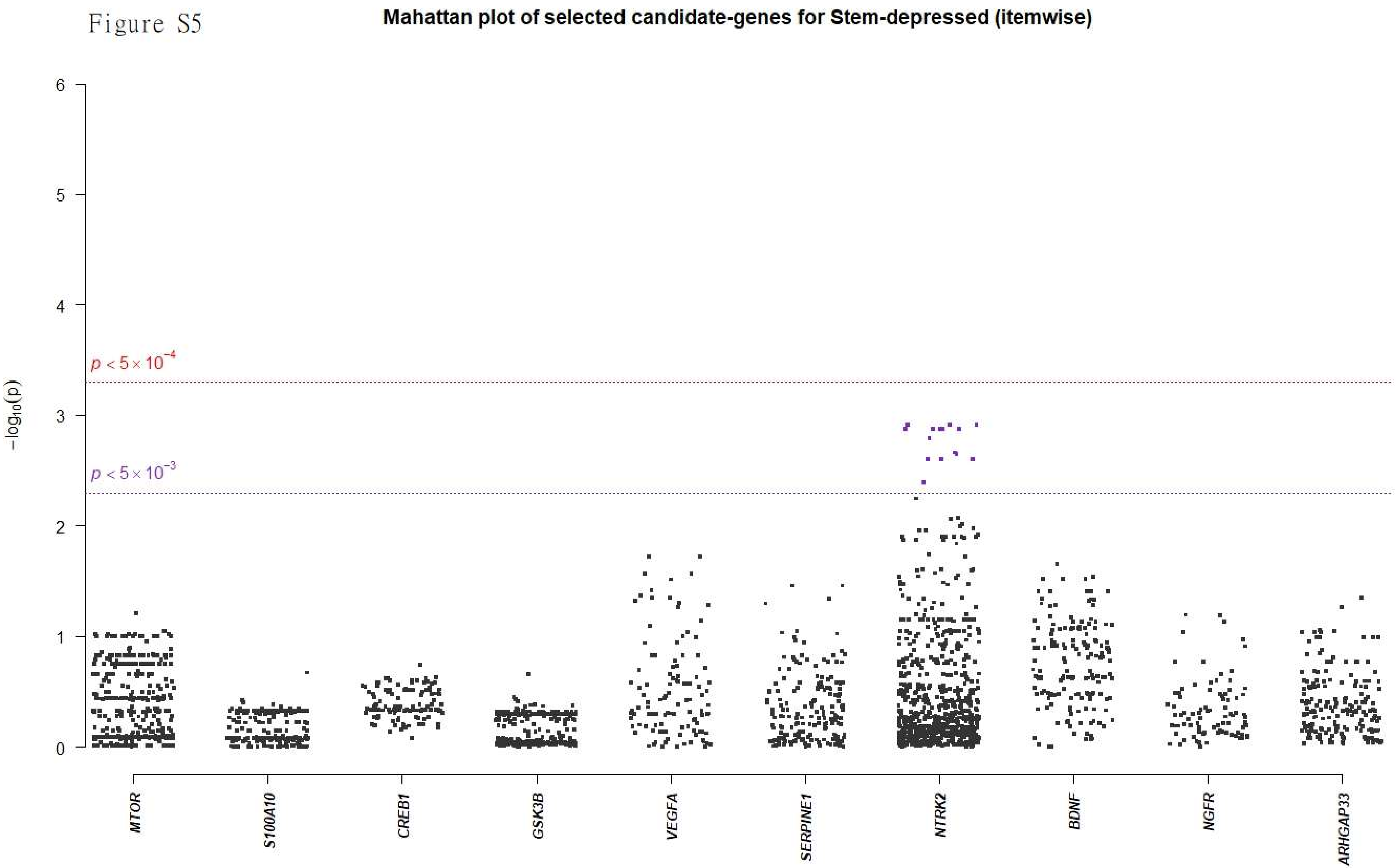

Mahattan plot of selected candidate-genes for Stem-depressed (itemwise)
6
5
4
3
2
1
0
−log10(p)
p < 5 × 10^−4
p < 5 × 10^−3
MTOR
S100A10
CREB1
GSK3B
VEGFA
SERPINE1
NTRK2
BDNF
NGFR
ARHGAP33

Figure S6

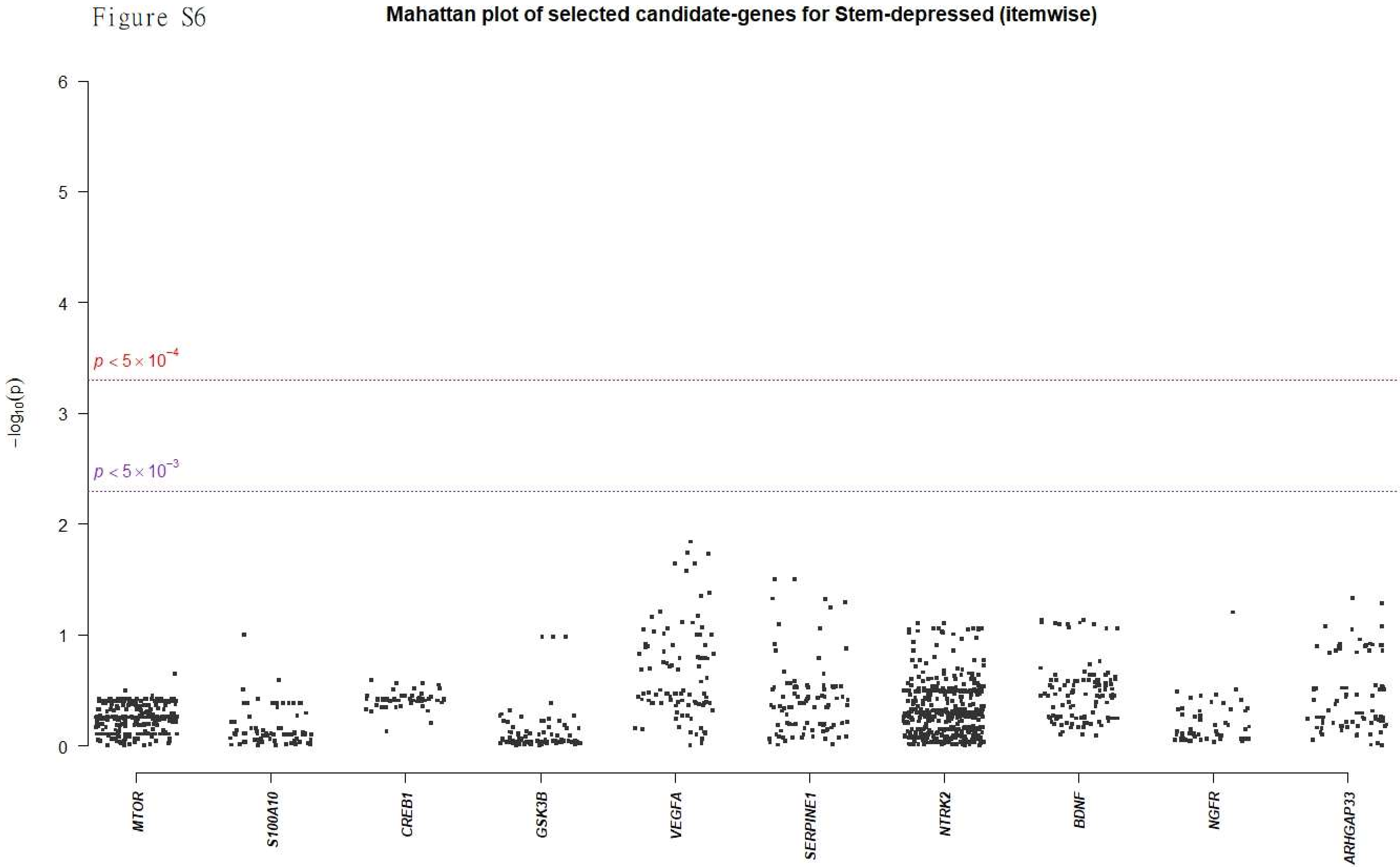

Mahattan plot of selected candidate-genes for Stem-depressed (itemwise)
6
5
4
3
2
1
0
$-\log_{10}(p)$
$p < 5 \times 10^{-4}$
$p < 5 \times 10^{-3}$
MTOR
S100A10
CREB1
GSK3B
VEGFA
SERPINE1
NTRK2
BDNF
NGFR
ARHGAP33
